# A commentary on ‘Incidence, risk factors, and outcomes of venous thromboembolism in patients undergoing surgery for retroperitoneal tumors: a propensity-matched retrospective cohort study’

**DOI:** 10.1097/JS9.0000000000000823

**Published:** 2023-10-26

**Authors:** Gang Wang, Ning Zhuo, Liu Zhichun

**Affiliations:** a Department of Rheumatology and Immunology, The Second Affiliated Hospital of Soochow University; b Department of Nephrology, The Affiliated Suzhou Hospital of Nanjing Medical University, Suzhou Municipal Hospital, Suzhou, People’s Republic of China

*Dear Editor,*

We read with interest the recent publication by Guo and colleagues on incidence, risk factors, and outcomes of venous thromboembolism (VTE) in patients undergoing surgery for retroperitoneal tumors: a propensity-matched retrospective cohort study^1^. The authors concluded that age, recurrence, and vascular resection are positively associated with VTE, which is associated with inferior overall survival^1^. We support and appreciate the authors’ work and agree with their conclusions, but we have some concerns about some of the details in the article.

First, the authors noted that patients with primary/recurrent retroperitoneal tumors who underwent surgery between January 2015 and December 2020 were included in this study^1^. The authors overlooked very important confounding factors: that is, since the outbreak of coronavirus disease 2019 (COVID-19), the severe acute respiratory syndrome coronavirus 2 (SARS-CoV-2) infection can have a significant impact on VTE in patients. Therefore, in this study, SARS-CoV-2 infection may have an impact on VTE. Multiple studies have shown that the risk of VTE increases significantly during SARS-CoV-2 infection, including in the venous and arterial systems^2^. At the same time, the risk of VTE in COVID-19 patients can persist from admission to up to 90 days after discharge^2^. COVID-19 infection may be a confounding factor in this study; therefore, additional analysis is necessary to explore the impact of SARS-CoV-2 infection on this study.

Second, studies have shown that the use of non-steroidal anti-inflammatory drugs (NSAIDs) is associated with a two-fold or greater increase in the risk of VTE^3^. A meta-analysis showed that the risk of VTE in NSAID users was significantly increased statistically^4^. A study showed that compared with commonly used NSAIDs, patients who started using tramadol had an increased risk of VTE within 1 year^5^. For patients with retroperitoneal tumors in this study, did they use analgesic drugs (such as tramadol and NSAIDs) to treat their cancer pain? If the author could add information in this regard, the results would be more satisfactory.

Third, the authors did not comprehensively assess risk factors that may influence the occurrence of VTE; for example, this study lacked detailed information on prophylactic anticoagulant use^1^. Thus, anticoagulant prophylaxis may be a hidden confounder. Other medications that affect VTE (e.g. oral contraceptives, anticoagulants, and antiplatelet drugs) were also missing in this study. Statins may have a protective effect against thrombosis. Moreover, the author did not systematically investigate whether there are congenital thrombotic disorders such as protein C and protein S deficiencies, Leiden V factor, antithrombin III deficiency, and acquired thrombophilia, such as antiphospholipid syndrome and nephrotic syndrome in this population^1^.

Finally, not all patients underwent lower limb venous imaging and computed tomography, pulmonary angiography, or ventilation/perfusion scanning. The study showed that ultrasound examination has significantly lower accuracy for isolated distal deep vein thrombosis (DVT) compared to proximal DVT, and some cases may actually be false positives. Moreover, it is known that some vascular ultrasound laboratories do not routinely scan the calf veins, including the soleus and gastrocnemius veins, which may limit the generalizability of the study results. Additionally, routine screening for VTE was not conducted for all included patients during the follow-up period, especially for subclinical thrombosis, intravascular microthrombi, and sporadic atypical site thrombosis, which may underestimate the true incidence of VTE events. For example, we cannot rule out the possibility that some asymptomatic isolated distal DVTs progress to proximal DVT or pulmonary embolism.

In conclusion, before these issues are clarified, this study’s findings should be interpreted cautiously.

## Ethical approval

Not applicable.

## Consent

Not applicable.

## Sources of funding

Not applicable.

## Author contribution

G.W. and N.Z.: wrote the paper; Z.L.: reviewed and edited the manuscript. Both authors had access to the data.

## Conflicts of interest disclosure

The authors declare that they have no conflicts of interest.

## Research registration unique identifying number (UIN)

Not applicable.

## Guarantor

Correspondence: Zhichun Liu, Department of Rheumatology and Immunology, The Second Affiliated Hospital, of Soochow University, Suzhou, People’s Republic of China; E-mail: zcliurheu@suda.edu.cn; ORCID: 0000-0001-6170-9601.

## Data availability statement

Data sharing is not applicable to this article as no new data were created or analyzed in this study.

## Provenance and peer review

My paper was not invited.
